# Call of the wild rice: *Oryza rufipogon* shapes weedy rice evolution in Southeast Asia

**DOI:** 10.1111/eva.12581

**Published:** 2018-01-11

**Authors:** Cynthia C. Vigueira, Xinshuai Qi, Beng‐Kah Song, Lin‐Feng Li, Ana L. Caicedo, Yulin Jia, Kenneth M. Olsen

**Affiliations:** ^1^ Department of Biology High Point University High Point NC USA; ^2^ Department of Ecology & Evolutionary Biology University of Arizona Tucson AZ USA; ^3^ School of Science Monash University Malaysia Selangor Malaysia; ^4^ Genomics Facility Monash University Malaysia Selangor Malaysia; ^5^ Ministry of Education Key Laboratory for Biodiversity Science and Ecological Engineering Department of Ecology and Evolutionary Biology Fudan University Shanghai China; ^6^ Department of Biology University of Massachusetts Amherst MA USA; ^7^ Dale Bumpers National Rice Research Center USDA‐ARS Stuttgart AR USA; ^8^ Department of Biology Washington University St. Louis MO USA

**Keywords:** adaptive evolution, dedomestication, introgression, population genomics, rice (*Oryza sativa*), weedy crop relatives

## Abstract

Agricultural weeds serve as productive models for studying the genetic basis of rapid adaptation, with weed‐adaptive traits potentially evolving multiple times independently in geographically distinct but environmentally similar agroecosystems. Weedy relatives of domesticated crops can be especially interesting systems because of the potential for weed‐adaptive alleles to originate through multiple mechanisms, including introgression from cultivated and/or wild relatives, standing genetic variation, and de novo mutations. Weedy rice populations have evolved multiple times through dedomestication from cultivated rice. Much of the genomic work to date in weedy rice has focused on populations that exist outside the range of the wild crop progenitor. In this study, we use genome‐wide SNPs generated through genotyping‐by‐sequencing to compare the evolution of weedy rice in regions outside the range of wild rice (North America, South Korea) and populations in Southeast Asia, where wild rice populations are present. We find evidence for adaptive introgression of wild rice alleles into weedy rice populations in Southeast Asia, with the relative contributions of wild and cultivated rice alleles varying across the genome. In addition, gene regions underlying several weed‐adaptive traits are dominated by genomic contributions from wild rice. Genome‐wide nucleotide diversity is also much higher in Southeast Asian weeds than in North American and South Korean weeds. Besides reflecting introgression from wild rice, this difference in diversity likely reflects genetic contributions from diverse cultivated landraces that may have served as the progenitors of these weedy populations. These important differences in weedy rice evolution in regions with and without wild rice could inform region‐specific management strategies for weed control.

## 1 INTRODUCTION

Crop domestication and agricultural weed evolution together represent two of the best documented forms of rapid evolution in plant species. Both of these processes are at play in the evolution of weedy crop relatives, which have recently gained attention as valuable systems for studying the genetic basis of rapid adaptation in agroecosystems (Vigueira, Olsen, & Caicedo, 2013). Weedy crop relatives are also increasingly recognized as long‐standing components of agroecosystems and integral contributors to crop evolutionary dynamics (Fénart, Arnaud, De Cauwer, & Cuguen, 2008; Li, Li, Jia, Caicedo, & Olsen, 2017; Roumet, Noilhan, Latreille, David, & Muller, 2013).

In the case of domesticated rice (*Oryza sativa* L.), conspecific weed strains occur in rice production areas worldwide, where they aggressively compete with crop varieties for nutrients and light. Weedy rice infestations can reduce harvests by more than 80% if left unchecked and are considered a primary constraint on rice productivity in the United States and other world regions. Weedy rice control is hindered by its close phenotypic similarity to its domesticated relative, especially at the vegetative stage, and by the potential for crop‐to‐weed movement of herbicide resistance alleles (Chen, Lee, Song, Suh, & Lu, 2004; Shivrain et al., 2009). Although both cultivated and weedy rice are predominantly self‐fertilizing, multiple instances of introgression of herbicide resistance alleles have been documented since the commercialization of herbicide‐resistant cultivars in the early 2000s (Busconi, Rossi, Lorenzoni, Baldi, & Fogher, 2012; Engku et al., 2016; Shivrain et al., 2007), indicating the potential for weed adaptation through crop‐to‐weed gene flow. Phenotypic characteristics of weedy rice include rapid growth and soil nutrient uptake; highly shattering seed that are easily dispersed into crop fields; strong seed dormancy, which allows seeds to remain viable in the seed bank for several years; and proanthocyanidin‐pigmented pericarps, a dormancy‐associated trait found in wild *Oryza* species (reviewed in Nadir et al., 2017).

As a conspecific weed of a genomic model crop species, weedy rice has provided a productive system for studying the dynamics of agricultural weed adaptation. Studies over the last two decades have characterized the population structure of weed strains and revealed independent weed origins in different world regions, with most weed strains closely related to domesticated rice (Akasaka, Ushiki, Iwata, Ishikawa, & Ishii, 2009; Cao et al., 2006; Cho, Chung, & Suh, 1995; Huang et al., 2017; Londo & Schaal, 2007; Song, Chuah, Tam, & Olsen, 2014). Weedy rice strains in the southern United States are among the genetically best characterized. A combination of analyses, including comparative QTL mapping (Qi et al., 2015), population genomics (Reagon et al., 2010), candidate gene studies (Gross et al., 2010; Reagon, Thurber, Olsen, Jia, & Caicedo, 2011; Thurber et al., 2010; Vigueira, Li, & Olsen, 2013), and selection scans with whole‐genome sequences (Li et al., 2017), has revealed that the two major strains present in US rice fields likely evolved in Asia through two independent episodes of dedomestication from cultivated rice. No wild *Oryza* species occur in North America, and there is minimal evidence for any direct role of wild populations in the evolution of these weed populations. The US weed strains are also characterized by low genetic diversity, consistent with population bottlenecks during their introduction from Asia; this most likely occurred as accidental contaminants of grain stocks (Reagon et al., 2010).

Examination of weedy rice in different regions of Asia can provide valuable points of comparison to the genetically well‐characterized US weed strains. Here, we focus on portions of Southeast Asia (Thailand, Vietnam, Cambodia, Malaysia, and Indonesia) and Northeast Asia (specifically South Korea). In the case of Southeast Asia, which represents one of the likely centers of early rice cultivation, three factors would be expected to contribute to more complex weed evolutionary dynamics than in other regions: (i) the presence of *Oryza rufipogon* Griff. (hereafter wild rice), the crop's wild progenitor which is outcrossing and interfertile with both cultivated and weedy rice (Majumder, Ram, & Sharma, 1997); (ii) a far greater diversity of crop varieties and landraces in this region, some of which could be contributing to the weed's evolution (Song et al., 2014); and (iii) the very rapid proliferation of weedy rice across this region in recent decades due to agricultural shifts away from hand‐transplanting of rice seedlings toward mechanized direct‐seeded rice cultivation (Chauhan, 2013; Sudianto et al., 2016). In contrast to Southeast Asian weeds, those in Northeast Asia are similar to US weeds in that they occur outside the geographical range of wild *Oryza* populations and the area of high crop varietal diversity.

Among these different factors that could shape weedy rice evolution, the presence or absence of wild rice populations could be particularly important for weedy rice adaptation. Some wild rice traits, such as freely shattering seed and persistent seed dormancy, would be expected to be highly adaptive if introgressed into weedy rice populations. In contrast, wild rice traits such as perenniality, sporadic seed production and prostrate plant architecture would all be expected to be maladaptive for survival in cultivated rice fields. Given this combination of potentially beneficial and maladaptive traits for weedy rice, one might expect differential evidence of wild‐to‐weed introgression in the specific genomic regions that would confer weed‐adaptive traits. Because rice is a genomic model species with a well‐annotated reference genome and molecularly well‐characterized domestication genes, evidence for such adaptive introgression can be explicitly examined using dense, genome‐wide SNP markers (Hufford et al., 2013). This genome‐wide approach can serve as a useful complement to recent candidate gene studies which have suggested adaptive introgression of wild rice alleles conferring shattering (*sh4*, Song et al., 2014) and seed dormancy (*Rc*, Cui et al., 2016) in some Malaysian weedy rice strains.

In this study, we used genome‐wide SNPs generated through genotyping‐by‐sequencing (GBS) to compare the genetic composition and evolution of weedy rice strains in Southeast Asia, Northeast Asia, and the United States. We specifically address the following questions: (i) How do weeds from these different world regions compare with respect to relationships to cultivated rice varieties and to wild rice? (ii) To what extent does wild rice hybridization shape the genetic composition of Southeast Asian weeds? (iii) Is there evidence that wild rice hybridization with weeds in Southeast Asia has led to the differential introgression of loci associated with weed‐adaptive traits?

## 2 METHODS

### 2.1 Sampling and genotyping

Rice seeds were obtained from the International Rice Germplasm Collection (IRGC), the United States Department of Agriculture (USDA), and from direct rice field collections in Malaysia (BK Song collections). Sampling included 133 weedy, 73 cultivated, and 34 wild rice accessions (Table 1 and Table S1) for a total of 240 accessions. Weedy rice sampling from outside the range of wild rice included 17 US accessions and 18 South Korean accessions. Southeast Asian weed accessions included 24 accessions from Vietnam, Thailand, Cambodia, and Indonesia (referred to collectively as VTCI), where wild rice commonly grows in close proximity to rice fields, as well as 74 weedy accessions from Malaysia, 22 of which were from Sabah (a Bornean state where wild rice does not occur) and 52 of which were from Peninsular Malaysia (where wild rice can be found near rice fields). Local cultivated and wild rice plants were also sampled from each of these regions where they occur. Sampling of cultivated rice accessions included all five major genetic subgroups within the crop: *indica* and *aus* (both within the traditionally recognized *indica* subspecies), and *aromatic*,* tropical japonica,* and *temperate japonica* (together composing the traditional *japonica* subspecies). Wild *Oryza* species included 25 *O. rufipogon*, two *Oryza barthii*, two *Oryza meridionalis*, and five *Oryza officinalis*; the first three are AA genome species that could potentially hybridize with cultivated and/or weedy rice in nature.

**Table 1:** Summary of 240 rice accessions used in this study including country of collection and type

Country	Weedy	Cultivated	Wild rice (*Oryza rufipogon)*	Other *Oryza* species
Malaysia	74	32	7	5
Indonesia	5	5	1	1
Thailand	10	8	5	
Vietnam	2	5	5	
Cambodia	7	4	3	
Southeast Asia Total	98	54	21	6
South Korea	18			
USA	17			
Other/unknown countries		19	4	3
Total	133	73	25	9

Seeds were germinated for each sampled accession in the greenhouse at Washington University, and leaf tissue was collected from young seedlings. DNA was extracted using DNeasy Plant DNA kits (QIAGEN) or a modified CTAB procedure (Doyle, 1991). DNA concentrations were determined using Qubit Fluorometric Quantification. Genotyping‐by‐sequencing was carried out on 1 μg of genomic DNA (100 ng/μl) at Cornell University's Genomic Diversity facility based on the methods outlined by Elshire et al. (2011). Briefly, each sample was digested with *Ape*KI followed by ligation of barcode and common adapters. Barcoded libraries were sequenced on an Illumina HiSeq2000 sequencer (Illumina Inc., San Diego, CA) with single‐end 100‐bp chemistry. Raw sequence data were processed using a standard TASSEL‐GBS pipeline (Bradbury et al., 2007). First, reads were filtered out if “N” was reported in the first 72 bases or a read did not contain a perfect match to any of the barcodes used in this study. Tags comprising fewer than five reads of the identical sequence were also discarded. All filtered tags were then aligned to the rice genome MSU 6.0 assembly (http://rice.plantbiology.msu.edu) using the Burrows–Wheeler alignment (BWA) tool (Li & Durbin, 2009), allowing a maximum of four mismatches and no gaps within 5 bp at the end of each read. SAMConverter was employed to convert SAM files to TagsOnPhysicalMap (TOPM) files, which were used to store information of the identified SNPs and small indels. Loci with more than 10% missing data and monomorphic data were discarded. After this filtering process, a total of 44,769 SNPs were retained for further analyses. Raw reads were submitted to the NCBI Short Read Archive (accession SRX576894).

### 2.2 Population structure, PCA, and nucleotide diversity analyses

Bayesian analysis of population structure was performed in fastSTRUCTURE (Raj, Stephens, & Pritchard, 2014), with *K* values varying from 1 to 10 and three replicates for each *K*. The Python script *chooseK.py*, incorporated with fastSTRUCTURE, was used to identify the *K* value that maximized the marginal likelihood. Principal components analysis (PCA) was performed using the *smartpca* software in the EIGENSOFT package (Patterson, Price, & Reich, 2006).

A sliding window analysis for each identified rice genetic group was performed to estimate relative nucleotide diversity between rice groups across the genome. SNPs were converted from HAPMAP format to VCF format using TASSEL (Bradbury et al., 2007), and average pairwise nucleotide diversity (π) was estimated with a window size of 300 kbp and a step size of 100 kbp for a total of 3,679 sliding windows in VCFtools (Danecek et al., 2011). The mean nucleotide diversity and variation were calculated and visualized in R. GBS data cover a reduced fraction of the genome, so nucleotide diversity estimates likely include only a fraction of the total SNPs present in any given window. These estimates will therefore be impacted by the use of GBS data; however, the relative nucleotide diversity estimates between rice groups should not be affected as the same set of markers are used in all rice groups.

### 2.3 Subpopulation structure of Malaysian weedy rice

Because a previous analysis of Malaysian weedy rice that we performed using simple sequence repeat (SSR) markers (Song et al., 2014) detected population structure that was not immediately evident in our fastSTRUCTURE analysis of all accessions (see Results), a separate fastSTRUCTURE analysis was carried out exclusively on Malaysian accessions. The previous study found that a phenotypic gradation in grain traits, specifically hull color and awn type (presence/absence), was correlated with a gradient of genetic composition in Malaysian weeds, from mostly crop‐like strains (straw‐hull, awnless) to mostly wild‐like strains (black‐hull, awned), with phenotypically intermediate and genetically admixed accessions in between the two extremes. We included the full set of 74 Malaysian weedy rice samples (22 Sabah and 52 Peninsular accessions), with phenotypic data incorporated from the previous study (Song et al., 2014; see also Sudianto et al., 2016), as well as 29 Malaysian crop landraces, four elite Malaysian cultivars, seven Malaysian wild rice accessions, and five accessions of the more distantly related species *O. officinalis*. The fastSTRUCTURE analysis of Malaysian samples was performed with the same parameter settings as for the full dataset.

### 2.4 Local ancestry estimation (HAPMIX)

Because population structure analysis of Southeast Asian weeds suggested evidence of ancestry from both cultivated and wild rice (see Results), we performed further analyses to identify the specific genomic regions showing evidence of introgression in weed populations. Analyses were performed using HAPMIX (Price et al., 2009), which infers ancestry across the genome of an admixed individual that is assumed to be derived from two proposed nonadmixed ancestor populations. Phased genotype data from the two nonadmixed ancestral populations and unphased data from putatively admixed descendants are required for HAPMIX modeling; therefore, only two‐way admixture can be simulated in this analysis (Price et al., 2009). Three weedy rice origin scenarios were investigated based on the results of population structure analyses: (i) weedy rice in VTCI (Vietnam, Thailand, Cambodia, Indonesia) derived by crossing between local wild rice and local crop landraces; (ii) weedy rice in Malaysia derived by crossing between local landraces and Malaysian elite cultivars; and (iii) weedy rice in Malaysia derived by crossing between local wild rice and Malaysian elite cultivars.

For the first scenario, VTCI wild rice (13 accessions) and landraces (15 accessions) were employed as the two putative parental populations, and the 19 weedy accessions from the same region were employed as the descendant population (Table S2). Indonesian samples were not included in this analysis because of insufficient wild rice collections in that country. To further investigate the role of wild alleles in the origin of wild‐like traits that are present in these weeds, six well‐studied domestication‐related genes that control weed‐adaptive traits were chosen to examine whether these genes or genomic regions were likely introgressed from wild accessions: *An‐1*, controlling awn development (Luo et al., 2013); *Bh4*, controlling hull color (Zhu et al., 2011); *sh4*, controlling grain shattering (Li, Zhou, & Sang, 2006); *qSW5*, controlling seed size (Shomura et al., 2008; Weng et al., 2008); *PROG1*, controlling prostrate versus erect growth (Jin et al., 2008; Tan et al., 2008); and *Rc*, controlling pericarp pigmentation (Sweeney, Thomson, Pfeil, & Mccouch, 2006). For each of these genes, VTCI weeds are characterized primarily by the phenotype found in wild rice. The genomic locations of these genes were verified using the latest rice reference genome assembly (IRGSP version 7).

For the second and the third admixture scenarios, we aimed to assess the extent to which three potential parental groups (elite cultivars, landraces, and wild rice) contributed to the Malaysian weedy rice genome. In the second scenario, the four Malaysian elite rice accessions and six Malaysian landrace accessions (which showed genetic similarity to weedy rice in population structure analyses) were employed as the two proposed parental populations. In the third scenario, we used seven Malaysian wild rice accessions in place of the landrace accessions used in the second scenario (Table S2). Although the actual parental accessions are unknown in each scenario, the use of these proposed parental accessions is arguably justified because they are geographically sympatric with the local weedy rice and genetically most similar based on our global ancestral estimation (see Results).

To convert SNP data from *ancestral map* format to the *EIGENSTRAT* format, which is required by HAPMIX, the CONVERTF function in the EIGENSOFT package (Patterson et al., 2006) was used. Positions with ambiguous and heterozygous SNPs were filtered out because their genotypes are not phased. After the filtering, three subsets with 12,387 SNPs, 25,461 SNPs, and 21,302 SNPs were left for each of the three respective admixture scenarios, with 597‐3,897 SNPs on each chromosome (Table S3). Analyses were run under a model assuming equal genetic contributions from both proposed ancestor populations; this assumption is consistent with population structure results. The number of generations since admixture was set to 10,000. This is most likely an overestimate, as the earliest origins of rice domestication likely date to no more than 10–12,000 years ago; however, HAPMIX results are robust to inaccurate estimations of time (Price et al., 2009), and the large estimate ensures that the time frame of weedy rice evolution is encompassed in the simulation. All of the remaining parameters used default settings. Results were visualized in R.

## 3 RESULTS

### 3.1 Population structure

**Figure 1:**
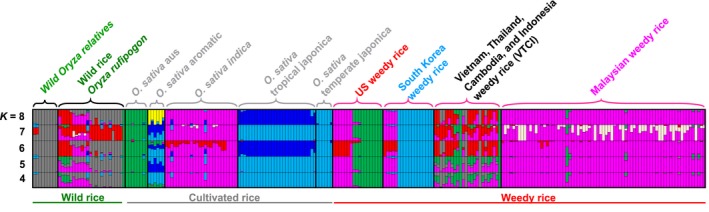
Population structure of 240 *Oryza* accessions based on 44,769 SNPs. FastSTRUCTURE results are shown from *K *=* *4–8. The maximized marginal likelihood indicates *K *=* *8 as the best fit grouping. Weedy rice from the United States, South Korea, and Malaysia group closely with cultivated rice varieties. Weedy rice from Vietnam, Cambodia, Thailand, and Indonesia group with both wild and cultivated rice accessions

Population structure analyses using fastSTRUCTURE of all rice accessions (Figure 1) indicates independent origins of weedy rice in the three sampled geographical regions. The maximized marginal likelihood values of fastSTRUCTURE outputs indicated *K *= 8 as the optimal grouping (Figure S1a,b). At this *K* value, the cultivated rice accessions are separated into their five previously described genetic subgroups: *indica* and *aus* varieties (within the *indica* subspecies), and *aromatic*,* tropical japonica,* and *temperate japonica* varieties (within the *japonica* subspecies). Results from *K *=* *4–7 are also presented for comparison. As found in previous studies (Li et al., 2017; Reagon et al., 2010), US weedy rice groups into two distinct subpopulations that are closely related to the two cultivated rice subgroups within the *indica* subspecies: *aus*‐like strains, corresponding to the previously described black‐hull awned (BHA) weeds, and *indica*‐like strains, corresponding to the previously described straw‐hull awnless (SH) weeds.

Like US weeds, the sampled South Korean weedy rice accessions also segregate into two distinct genetic groups. For these samples, however, one group is *indica*‐like while the other is grouped with *japonica* rice, specifically the *temperate japonica* varieties cultivated primarily in Northeast Asia (Figure 1). This evidence for *temperate japonica*‐derived South Korean weeds is consistent with other reports of *japonica‐*like weedy rice in temperate east Asia (Cho et al., 1995; He, Kim, & Park, 2017). Neither US nor South Korean weedy rice strains show evidence of population components characteristic of wild rice, which is consistent with origins from dedomesticated crop varieties in these regions occurring outside the geographical range of wild rice.

Patterns observed in the fastSTRUCTURE analysis for US and South Korean weeds are also evident in the principal components analysis (PCA); US weeds are clustered with *indica* and *aus* rice varieties, and South Korean weeds are clustered with *indica* and *temperate japonica* rice (Figure S2a,b). The *indica‐*like US and South Korean weeds are largely nonoverlapping in the PCA, which potentially suggests independent origins for these temperate‐adapted *indica*‐like weed strains.

In contrast to the US and South Korean weed strains, weedy rice samples from VTCI share substantial genetic similarity with wild *Oryza* samples (Figure 1; Figure S2). This is especially evident in the fastSTRUCTURE output. Two of the genetic subpopulations that are highly represented in the VTCI weeds at *K *= 8 are well represented in wild accessions and absent in domesticated rice (red and gray components in Figure 1). The VTCI weeds also contain *indica*‐ and *aus*‐like genetic backgrounds, which are present in both domesticated and wild rice. *Indica* rice is widely cultivated in Southeast Asia, and the *indica* component is consistent with local *indica* crop ancestry. In contrast, cultivation of *aus* rice varieties is restricted to northern regions of the Indian subcontinent and is absent in Southeast Asia. As such, the *aus*‐like component may be more likely to reflect weed descent from *aus*‐like wild rice than from cultivated *aus* ancestors. Overall, the combination of crop‐like and wild‐like genetic components in VTCI weeds suggests an origin through hybridization between cultivated rice and wild rice growing in close proximity to rice fields.

**Figure 2:**
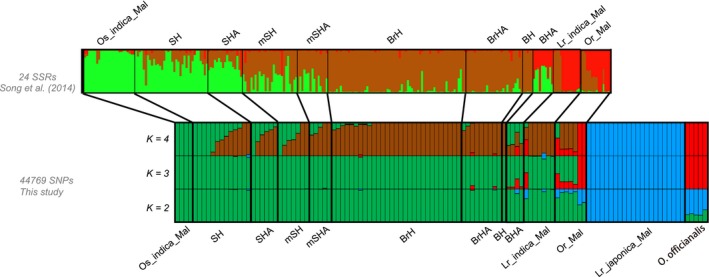
Fine‐scale population structure of Malaysian wild, cultivated, and weedy rice accessions. The top panel represents STRUCTURE results extracted from Song et al. (2014) based on 24 SSR markers. Below the top panel is the FastSTRUCTURE output of the same accessions using 44,769 SNPs. FastSTRUCTURE results are shown for *K *=* *2–4. Malaysian weed abbreviations: SH, straw‐hull awnless; SHA, straw‐hull awned; mSH, morphologically intermediate weed form between SH and BrH; mSHA, morphologically intermediate weed form between SHA and BrHA; BrH, brown‐striped hull awnless; BrHA, brown‐striped hull awned; BH, black‐hull awnless; BHA, black‐hull awned. Other abbreviations: Os, *Oryza sativa*; Mal, Malaysia; Lr, landrace; Or, *Oryza rufipogon*

Malaysian weed samples are genetically distinct from the other Southeast Asian weeds. (Figure 1; Figure S2). In both the fastSTRUCTURE analysis and PCA, Malaysian strains group primarily with *indica* crop varieties. This pattern differs from what we observed in an earlier study that examined Peninsular Malaysian weedy rice using a panel of polymorphic SSR markers (Song et al., 2014). That analysis revealed a gradation in the genetic composition of weed strains, from predominantly crop‐like, with a particularly close relationship to elite Malaysian *indica* cultivars, to predominantly wild‐like. The genetic gradation also corresponded to a phenotypic gradation in grain characteristics, from most crop‐like (straw‐hull awnless, SH) to most wild‐like (black‐hull awned, BHA). To assess whether this previously documented genetic heterogeneity could be detected with the GBS data, we performed a fastSTRUCTURE analysis focused exclusively on Malaysian samples; Figure 2 presents the results in comparison with the previously reported results of Song et al. (2014). Although marginal likelihood values suggest that the optimal *K* value was 3 (Figure S3), we also included fastSTRUCTURE results of *K *= 2 and 4 to avoid biased interpretations. (Membership assignments at *K *= 5 are associated with a secondary delta‐*K* peak but were largely indistinguishable from *K *= 4 and are not presented). At *K *= 2 and *K *= 3, Malaysian *japonica* landraces and wild *O. officinalis* are distinct from the remaining accessions. When *K* value is increased to 4, the Malaysian weeds show the pattern observed by Song et al. (2014), with a genetic gradient in the proportions of two subpopulations that correlates with the phenotypic gradient from crop‐like to wild‐like weeds. As observed in the earlier study, lighter hulled weeds group more with Malaysian elite *indica* rice (Os_*indica*_Mal in Figure 2), while darker hulled weeds group more with Malaysian landrace *indica* rice and Malaysian *O. rufipogon* (Lr_*indica*_Mal and Or_Mal in Figure 2). The BHA weed morphotype has a pattern of genetic admixture as found by Song et al. (2014). Together, these findings support a prominent role of Malaysian elite *indica* cultivated rice in the origin of local weedy rice, which is consistent with the previous study by Song et al. (2014).

### 3.2 Genetic diversity

**Figure 3:**
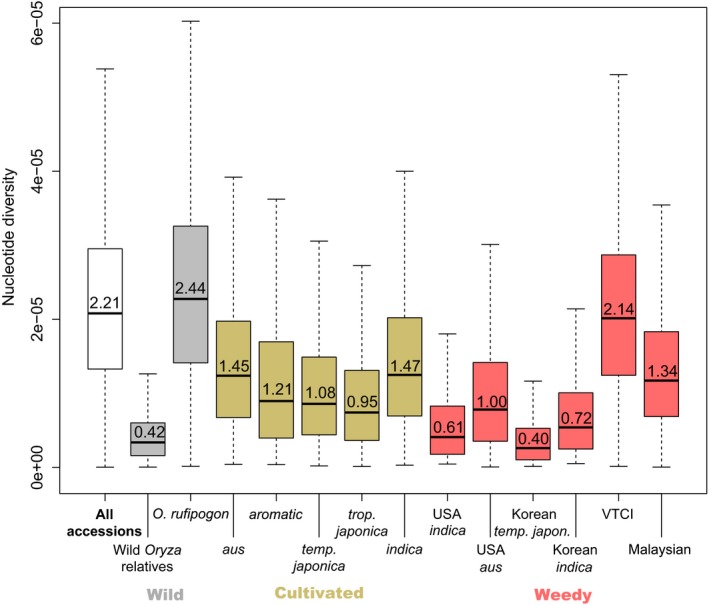
Box plots of nucleotide diversity from sliding windows across the rice genome for each *Oryza* group. Boxes represent 25th to 75th percentile and mean values are indicated inside each box. Whiskers are high and low values from 3,679 sliding windows. Groups include wild rice species (gray boxes), cultivated rice varieties (gold boxes) and weedy rice populations (pink boxes). VTCI, weedy rice from Vietnam, Thailand, Cambodia, and Indonesia

In order to evaluate genome‐wide nucleotide diversity, 3,679 sliding windows across the rice genome (on average 36 SNPs per window) were surveyed (Figure 3). The overall mean nucleotide diversity for all accessions was 2.21 × 10^−5^. Nucleotide diversity is highest in wild rice samples (*O. rufipogon*, 2.44 × 10^−5^) and reduced in each of the five cultivated rice groups (ranging from 0.95 × 10^−5^ for *tropical japonica* to 1.47 × 10^−5^ for *indica*). This pattern is consistent with the previously documented domestication bottleneck in cultivated rice (Caicedo et al., 2007). US and South Korean weedy rice have reduced nucleotide diversity when compared to their putative cultivar ancestors (US *indica* type: 0.61 × 10^−5^; US *aus* type: 1.00 × 10^−5^; South Korea *temperate japonica* type: 0.40 × 10^−5^; South Korea *indica* type: 0.72 × 10^−5^). These patterns are consistent with a second population bottleneck during dedomestication of weedy rice from cultivated rice (see Reagon et al., 2010). In contrast, weedy rice from VTCI has nucleotide diversity levels that are only slightly lower than the levels found in *O. rufipogon* (2.14 × 10^−5^). These high levels of nucleotide diversity could be driven by genetic input from multiple sources into these weeds, particularly *indica* cultivated rice and *O. rufipogon*. Malaysian weeds show a slight reduction in genetic diversity when compared to *indica* cultivars (1.34 × 10^−5^).

### 3.3 Admixture characterization

To quantitatively describe the degree of admixture in different weedy rice populations revealed by the fastSTRUCTURE analysis, we adopted a threshold in which individuals whose genome is composed of <80% of their dominant genomic component are considered as resulting from admixture. We then calculated the percentage of admixed samples using this threshold. We used the fastSTRUCTURE results from the analysis of all samples (Figure 1) except for the Malaysian weedy population, where we used the *K *=* *4 results from the Malaysian subpopulation fastSTRUCTURE results (Figure 2). Based on these criteria, none of the weed samples from the United States and South Korea reached the threshold for admixture. In contrast, 66.7% of VTCI weeds and 28.4% of Malaysian weeds samples were identified as admixed. The difference between these two Southeast Asian regions may partially be due to the fact that Malaysian samples from Sabah (representing 22 of the 77 Malaysian samples) are from outside the region of sympatry with wild rice and therefore would not have opportunities for wild‐to‐weed introgression.

**Figure 4:**
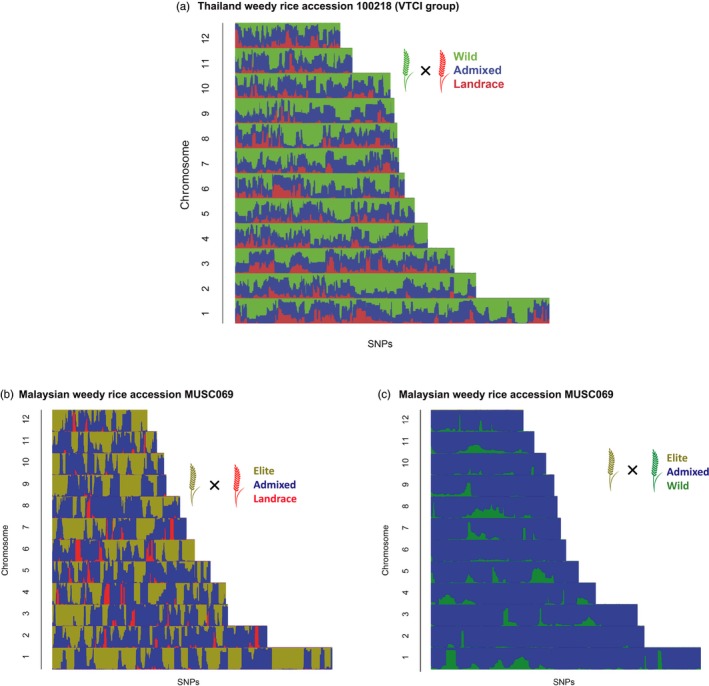
Bar plots of HAPMIX results. (a) Thai weedy accession 100218 (representing weeds from Vietnam, Thailand, Cambodia, and Indonesia) under landrace × wild admixture model, (b) Malaysian weedy accession MUSC069 under the elite × landrace model, and (c) Malaysian weedy accession MUSC069 under the elite × wild rice model. Each horizontal bar represents one chromosome with SNPs along their length. The color for each SNP represents the probability of that SNP originating from one of the parent populations (green, wild; red, landrace; or gold, elite cultivar) or the probability of admixture between the two parents at that site (blue)

As a complement to the quantitative measures of admixture, we used HAPMIX analysis to provide finer‐scale genomic resolution in identifying specific genomic regions involved in introgression. We observed consistent patterns among weedy samples within each of the three tested scenarios; we therefore present one representative weed accession from VTCI (Thai accession 100218) and one from Peninsular Malaysia (accession MUSC069) to illustrate the observed patterns (Figure 4a–c). For the VTCI samples, there is a higher inferred level of contribution from wild rice (green) than from local landraces (red) (Figure 4a). Using a criterion of >80% ancestry to define genomic regions with a dominant contribution from a single ancestor, we found that 5.4% of the VTCI genomes are dominated by wild rice, with <1% showing a predominant inferred contribution from local landraces (Figure S4). Notably, all six investigated weediness candidate genes (*An‐1*,* Bh4*,* sh4*,* qSW5*,* PROG1*, and *Rc*) were located within a genomic region which was dominated by wild rice alleles (Figure S5). Consistent with these patterns, seed phenotyping confirmed that all of the 19 tested Southeast Asian weedy rice accessions have dark hulls and awn presence, traits conferred by wild alleles of *An‐1* and *Bh4*. Thus, the patterns of wild rice ancestry across the genome are consistent with introgression of alleles conferring weed‐adaptive traits.

For Malaysian weedy rice, HAPMIX results indicate a smaller contribution of *indica* landrace when compared to *indica* elite cultivars under the *indica* elite × *indica* landrace scenario (Figure 4b). However, most of the genome shows patterns of admixture of elite *indica* cultivars and *O. rufipogon* under the *indica* elite × *O. rufipogon* scenario (Figure 4c). When we applied the same genomic dominance criteria as above under the *indica* elite × *indica* landrace scenario, 32.4% of the Malaysian weedy rice genome regions were dominated by *indica* elite alleles, 38.8% were dominated by admixture of the two parental populations, and 2.0% were dominated by *indica* landrace alleles. Under the *indica* elite × *O. rufipogon* scenario, 79.0% of the Malaysian weedy rice genome regions were dominated by admixture of the two parental populations and 4.5% were dominated by wild rice alleles (Figure S4). Thus, under either scenario, elite *indica* cultivars are inferred to have a pervasive genetic contribution across the Malaysian weed genome.

## 4 DISCUSSION

**Figure 5:**
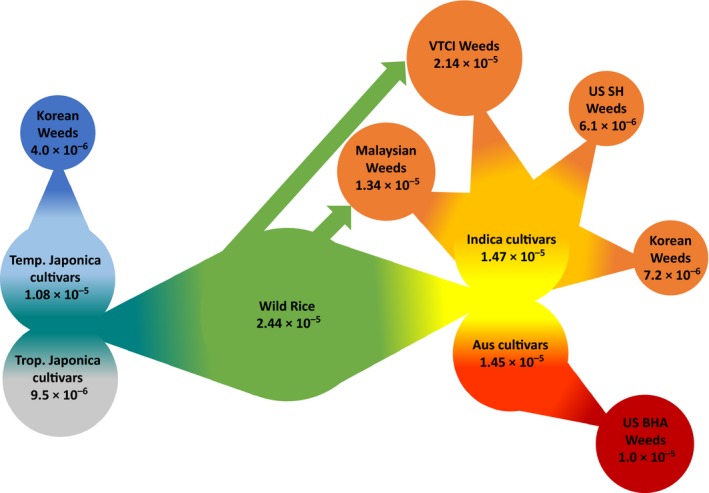
Current hypothesis of relationships between wild, cultivated, and weedy rice included in this study. Circles represent each group with connected triangles that represent population bottlenecks that lead to establishment of the new group. Green arrows represent introgression from wild rice into weedy populations after establishment. Average nucleotide diversity (π) is indicated for each group

Weedy rice provides a valuable model to study rapid evolution and to test the importance of introgression from crops and their wild relatives in this process. In this study, we used more than 40,000 genome‐wide SNPs to study the origin of weedy rice in five Southeast Asian countries, where wild rice co‐occurs with cultivated rice, and compared the observed genetic patterns with weedy rice origin in locations where wild rice is not present (Sabah state of Malaysia, USA, and South Korea). Our population genetic analyses reveal two key mechanisms of weedy rice evolution: (i) adaptive introgression from wild rice, which has played an important role in weed evolution from Southeast Asia, and (ii) dedomestication, which is the major mechanism of weedy origination in the USA and South Korea. Figure 5 illustrates our current model of weedy rice evolution from these different world regions, including proposed population bottlenecks leading to new weedy rice populations in different world regions (dedomestication) and subsequent introgression of alleles from wild rice in Southeast Asian populations.

### 4.1 Contributions of wild and cultivated rice to weedy rice evolution

Weedy rice strains from different world regions have originated independently from one another. In nearly all cases, there is some genetic contribution of cultivated rice to the ancestry of weedy rice populations. However, the contribution of wild rice to weedy rice ancestry varies widely. US and South Korean weeds have no indication of wild rice contributions, and dedomestication events from cultivated rice seem to be responsible for the origin of these weeds. Population bottlenecks associated with dedomestication events are evident in our nucleotide diversity reductions, when compared to cultivated relatives, for each of these weedy populations. *Oryza rufipogon* has contributed to weedy rice evolution in Peninsular Malaysia, Vietnam, Cambodia, Thailand, and Indonesia. Our data suggest that in regions where wild rice is present, one can expect there to be introgression of wild rice alleles into weedy rice populations. This could occur by two mechanisms: (i) weed evolution through hybridization of crop and wild populations and (ii) weed evolution through dedomestication followed by introgression from wild rice. While it is not possible for us to definitively determine which of these scenarios is correct for the Southeast Asian weeds examined in this study, in both cases wild rice has likely contributed adaptive alleles to weedy rice populations through introgression. This may result in weedy rice populations that are more difficult to control in Southeast Asia compared to populations where wild rice is not contributing alleles.

Weedy rice has evolved from different lineages of domesticated rice. *Indica‐*type weeds are most common; there is evidence of *indica* backgrounds in weedy rice found in the United States, South Korea, and all represented countries of Southeast Asia (Figures 1 and 5). Interestingly, it seems that *indica*‐like weeds from Malaysia are genetically more similar to US *indica*‐like weeds than weeds from other parts of Southeast Asia or South Korea (PCA; Figure S2). This could point to a shared common ancestor of Malaysian and US weeds. That conclusion is not supported by previous findings based on SSR markers (Song et al., 2014), a difference that could potentially reflect the higher‐resolution genomic sampling in the present study. However, *indica* cultivars also cluster with US and Malaysian weedy rice in our PCA (Figure S2), so we cannot rule out independent dedomestication events leading to these two weedy rice populations. The contribution of *aus* genes to weedy rice in Southeast Asia is smaller than that of *indica*, but is still present. Given the absence of *aus* rice cultivation in Southeast Asia, this contribution could most plausibly reflect introgression from *aus*‐like wild rice rather than cultivated *aus* varieties.

A subset of South Korean weedy rice is of *temperate japonica* origin. Weedy rice found in California (USA), which was not included in this study, is also of *temperate japonica* origin (Kanapeckas et al., 2016). It would be interesting to investigate whether these two weedy rice populations evolved independently from one another or reflect a single dedomestication event. To our knowledge, there is no evidence of weedy rice evolution from *tropical japonica* or *aromatic* rice varieties.

### 4.2 Standing variation versus adaptive introgression

South Korean and US weedy rice populations do not show evidence of introgression of alleles from wild rice since their origin. Therefore, weedy adaptive traits that are found in these populations are likely derived from standing genetic variation in the cultivated rice ancestral population. Previous studies have found standing variation in cultivar ancestors to be a contributing factor in weedy traits. The functional *Rc* allele that results in red‐colored pericarps in wild rice is present in some cultivated rice varieties that are the likely source of this allele in weedy rice from the United States (Gross et al., 2010). Genetic bottlenecks during dedomestication could contribute to fixation of rare alleles from cultivated varieties in weedy populations. In a worldwide survey of cultivated and wild rice, one *aus* cultivar shared a functioning *Bh4* allele, which underlies the black‐hull phenotype, with the fixed allele of BHA weedy rice from the United States (Vigueira, Li et al., 2013). However, standing variation does not seem to be the most likely source of all weed‐adaptive traits in US weedy rice; for example, the *sh4* allele that underlies loss of seed shattering in cultivated rice is also fixed in US weedy rice (Thurber et al., 2010). The gain of seed shattering in these weeds may thus be due to recombination of standing genetic variation in the weeds or new mutations since weed establishment. Unique genetic QTLs are responsible for shattering in BHA and SH weeds from the United States (Qi et al., 2015), suggesting different underlying genetic mechanisms in the re‐emergence of shattering.

Our genomic analysis of weedy rice from Southeast Asia paints a different picture from that of US weeds. Wild rice has contributed weedy adaptive alleles to weedy populations from Malaysia, Thailand, Vietnam, Cambodia, and Indonesia. Wild rice is the inferred contributing parent for genomic regions that house the six candidate weedy genes we tested (Figure S5), specifically *An‐1,* controlling awn development (Luo et al., 2013); *Bh4*, controlling hull color (Zhu et al., 2011); *sh4*, controlling grain shattering (Li et al., 2006); *qSW5*, controlling seed size (Shomura et al., 2008; Weng et al., 2008); *PROG1*, controlling prostrate versus erect growth (Jin et al., 2008; Tan et al., 2008); and *Rc*, controlling pericarp pigmentation (Sweeney et al., 2006). The wild rice contributions at all six of these regions suggest that this pattern is due to adaptive introgression rather than random, neutral introgression of portions of the genome.

One pattern that emerges from all of the weedy rice strains examined here is that to become weedy, rice apparently requires some genomic background from cultivated rice mixed with adaptive weedy alleles that are gained either through standing variation, new mutations, or adaptive introgression from wild rice. In regions where reproductively compatible wild rice is present, it appears that adaptive introgression from wild relatives is an effective route to acquisition of adaptive alleles. A more complete sampling of weedy rice, wild rice, and cultivars from Southeast Asia would be beneficial to untangle exactly which genomic regions are cultivar‐like and which are wild‐like across weedy populations. These regions, if shared across populations, would presumably be important to weedy rice establishment or maintenance. It would also be interesting to track this over time as management of rice fields in Southeast Asia changes from traditional practices to more mechanized farming.

### 4.3 Weedy rice management implications

Weedy rice populations have undergone a massive increase in regions of Malaysia and Thailand as a result of agronomic shifts toward industrialized rice production that rely on mechanized direct seeding of rice fields (Chauhan, 2013; Sudianto et al., 2016). Traditional rice farming in this region has relied on hand‐transplanting of paddy‐grown rice seedlings into prepared, flooded fields. While extremely labor‐intensive, that method provides ample opportunities for hand‐weeding of rogue weed seedlings. In contrast, direct seeding reduces opportunities for weeding and increases risks of weed seed dissemination between fields through shared farm equipment (Chauhan, 2013; Nadir et al., 2017). In Thailand, agricultural changes in the last decade away from direct seeding toward mechanized planting of seedlings have proved an effective weed control strategy (S. Jamjod, Chiang Mai Univ., pers. comm.). As the cultivation of herbicide‐resistant rice planting has become more popular with Malaysian farmers in the last decade, the problem of herbicide‐tolerant weedy rice has concurrently arisen (Engku et al., 2016; Ruzmi, Ahmad‐Hamdani, & Bakar, 2017). This causes additional strain on the rice industry, particularly in regions where herbicide‐tolerant rice cultivation has not been widely adopted.

Southeast Asian weedy rice is marked by much higher levels of genetic diversity than weedy rice from the other world regions sampled for this study. This high level of diversity as well as the continued potential for genetic introgression from both wild and cultivated rice in the region will likely make management of weedy populations extremely difficult. Although weed control in Thailand is showing promise over the last decade, the genetic potential for adaptation to these new practices is quite high in Thailand's weeds. Given our findings, management strategies that include control of wild rice populations in closest proximity to cultivated fields may be beneficial to weed control, as this could reduce the continued movement of adaptive alleles from wild rice into weedy populations. The high levels of genetic diversity in weedy rice from Southeast Asia further suggest that management in those regions should not rely solely on a single method (such as herbicide treatment), as the potential for rapid adaptive evolution is extremely high. Regardless of approach, careful monitoring of weedy populations during any shift in agricultural practices should be used to ensure the continued effectiveness of weed management strategies.

## DATA ARCHIVING STATEMENT

Data from this study are archived in the NCBI Short Read Archive (accession SRX576894).
