# Supplementary figures and images for: Call of the wild rice: Oryza rufipogon shapes weedy rice evolution in Southeast Asia
Source: Evol Appl. 2018 Jan 11;12(1):93–104. doi: 10.1111/eva.12581 (PMC6304679; doi:10.1111/eva.12581)

(a)

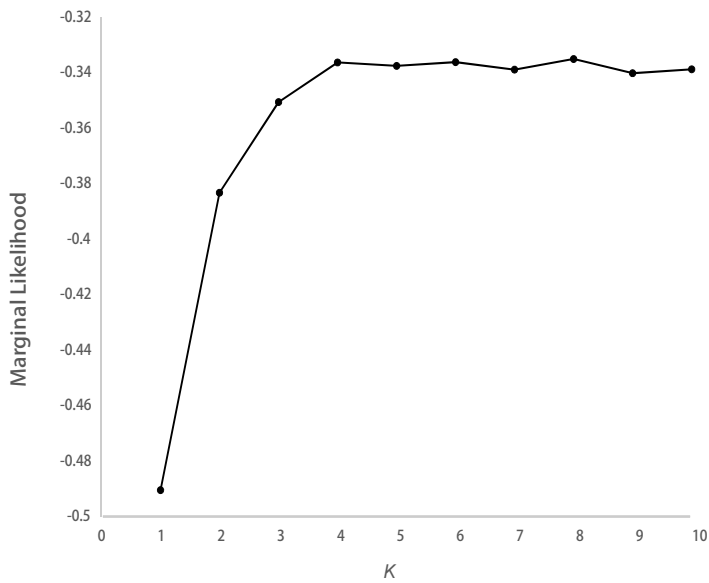

(b)

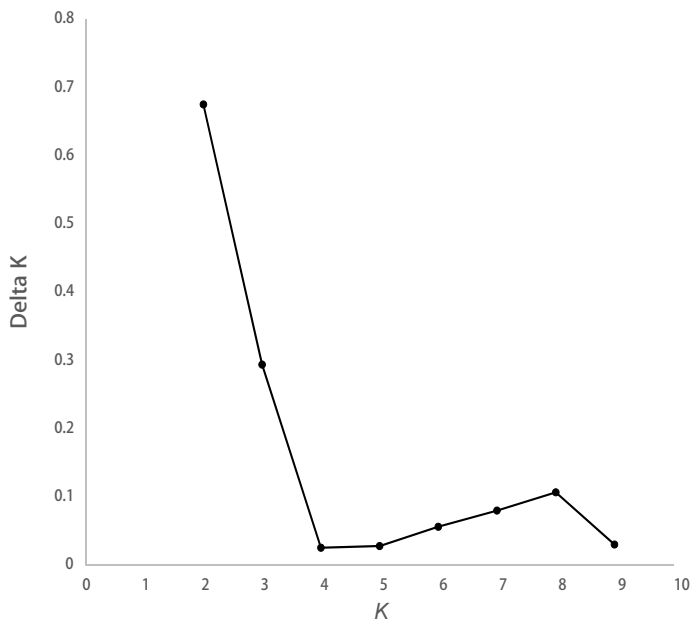

Supplement: Supplementary file 1 [file EVA-12-93-s001.pdf]

(a)

Wild and cultivated rice

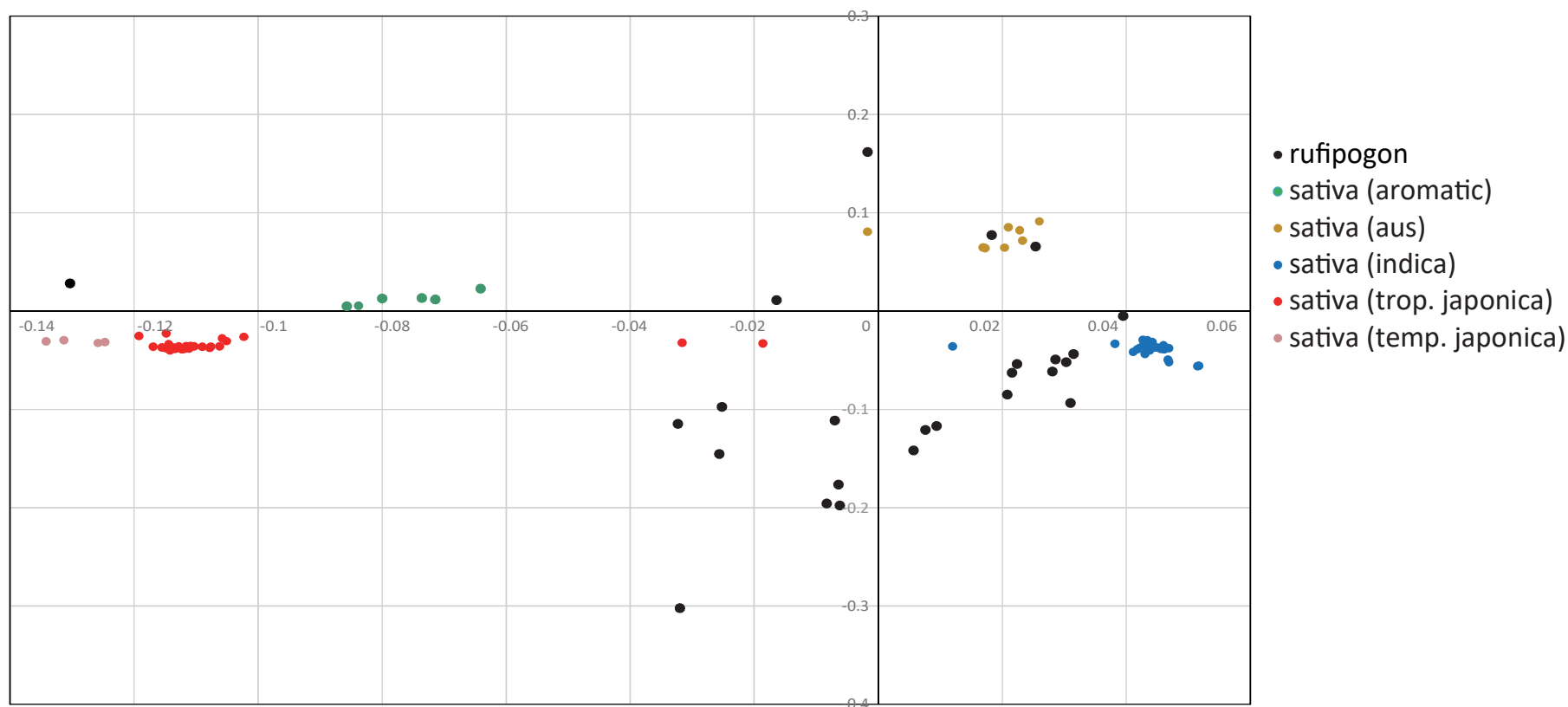

(b)

Wild, cultivated and weedy rice

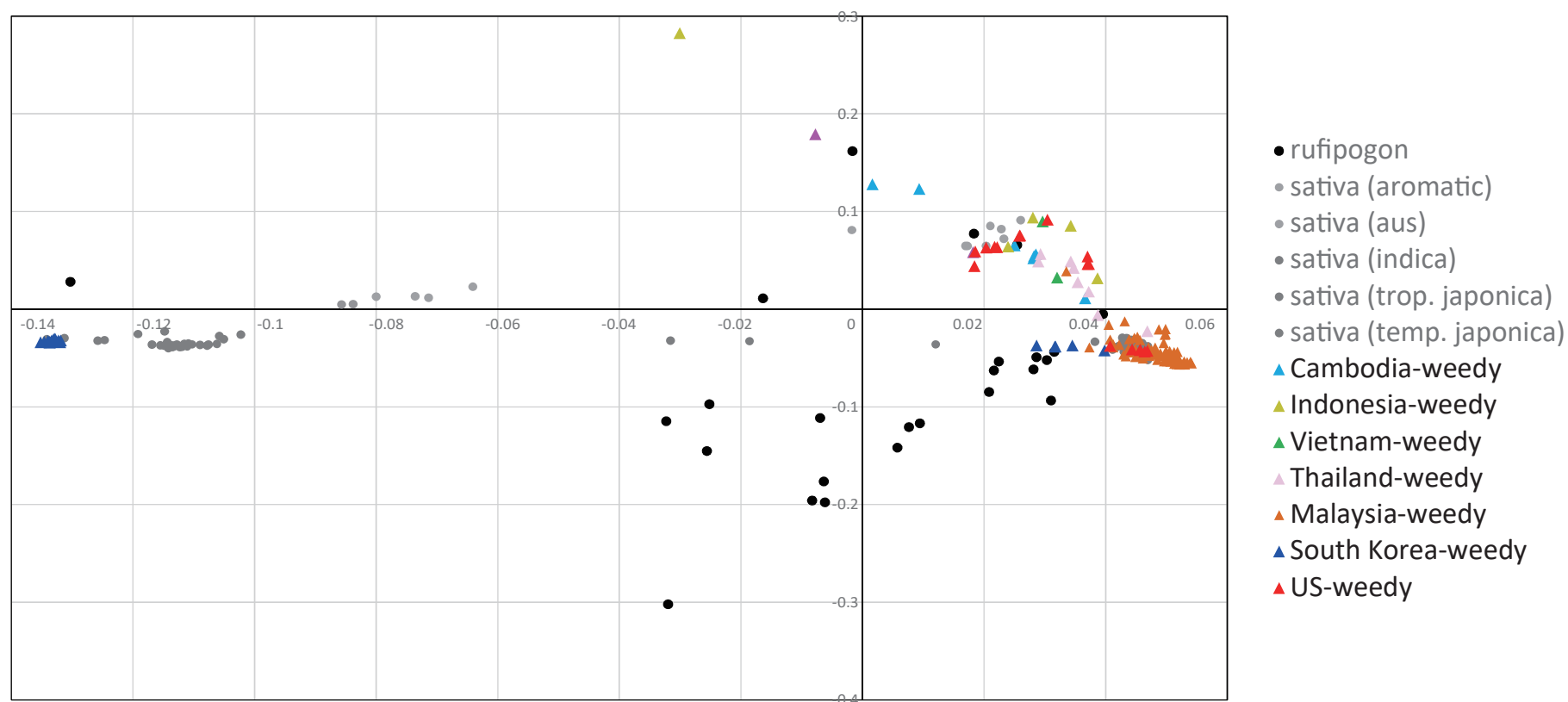

Supplement: Supplementary file 2 [file EVA-12-93-s002.pdf]

(a)

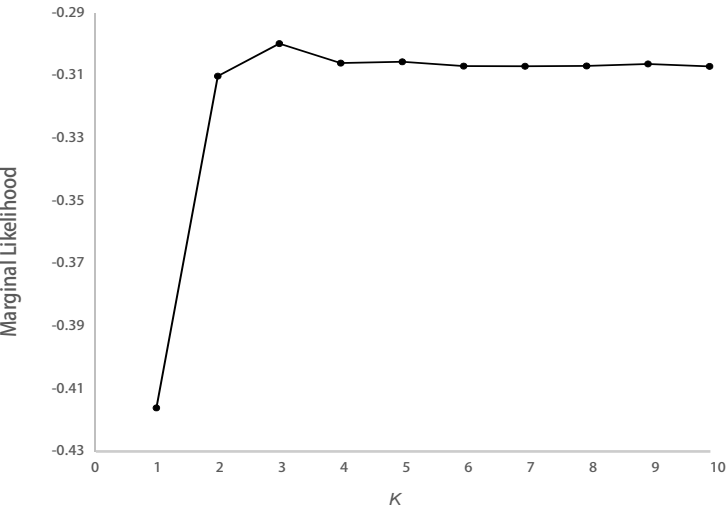

(b)

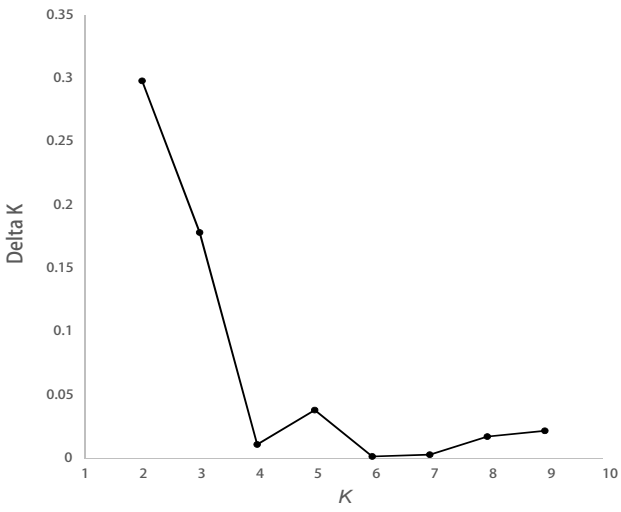

Supplement: Supplementary file 3 [file EVA-12-93-s003.pdf]

(a) VTCl: wild x landrace

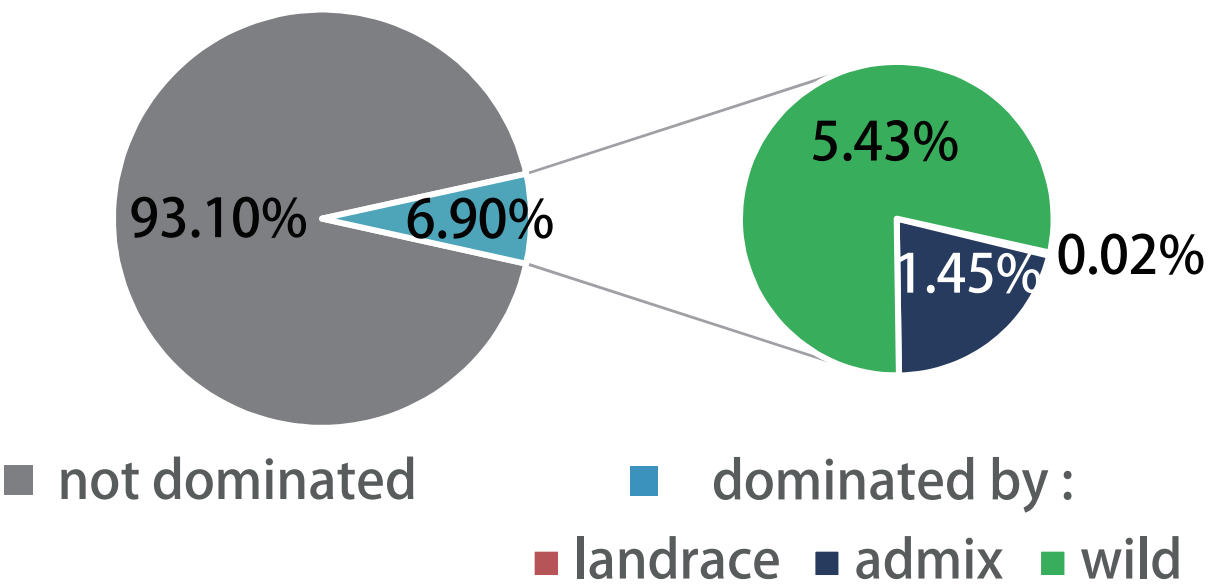

(b) Malaysia: landrace x elite

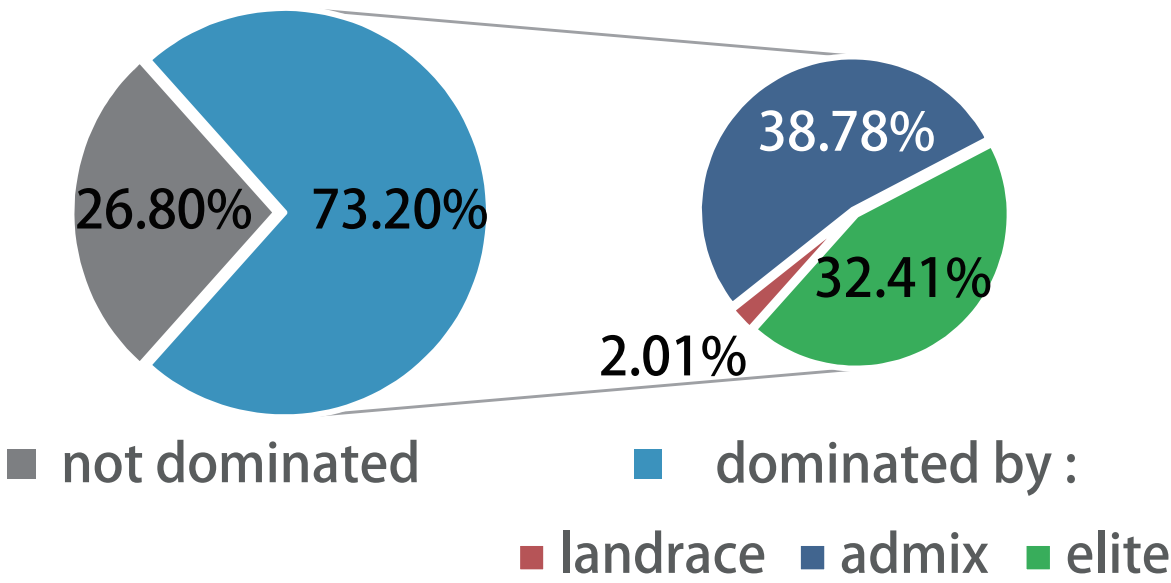

(c) Malaysia: wild x elite

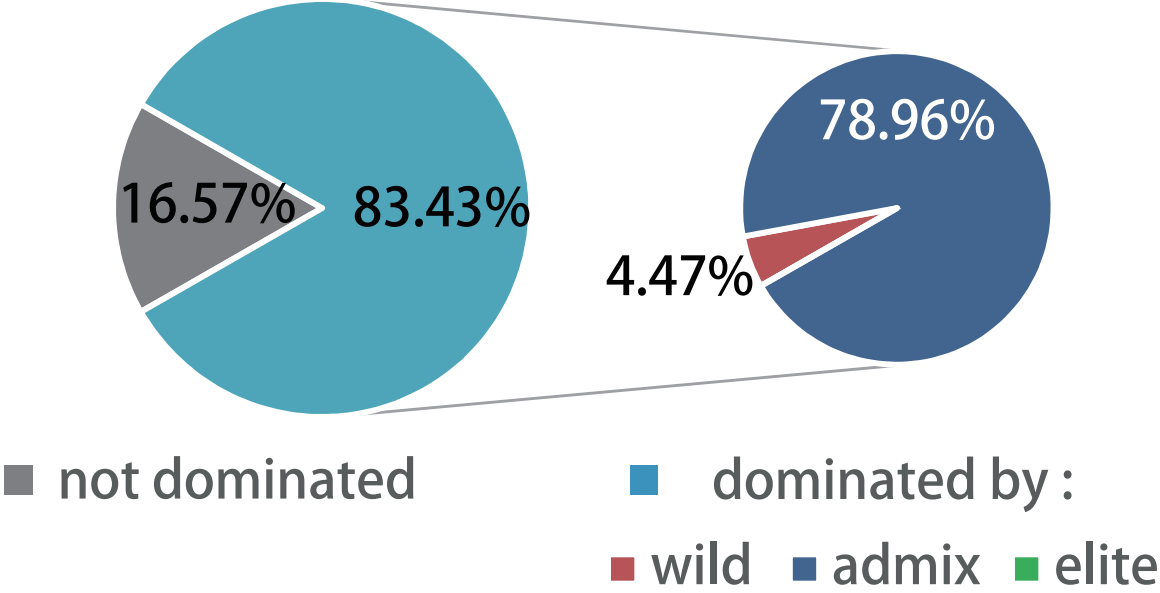

Supplement: Supplementary file 4 [file EVA-12-93-s004.pdf]

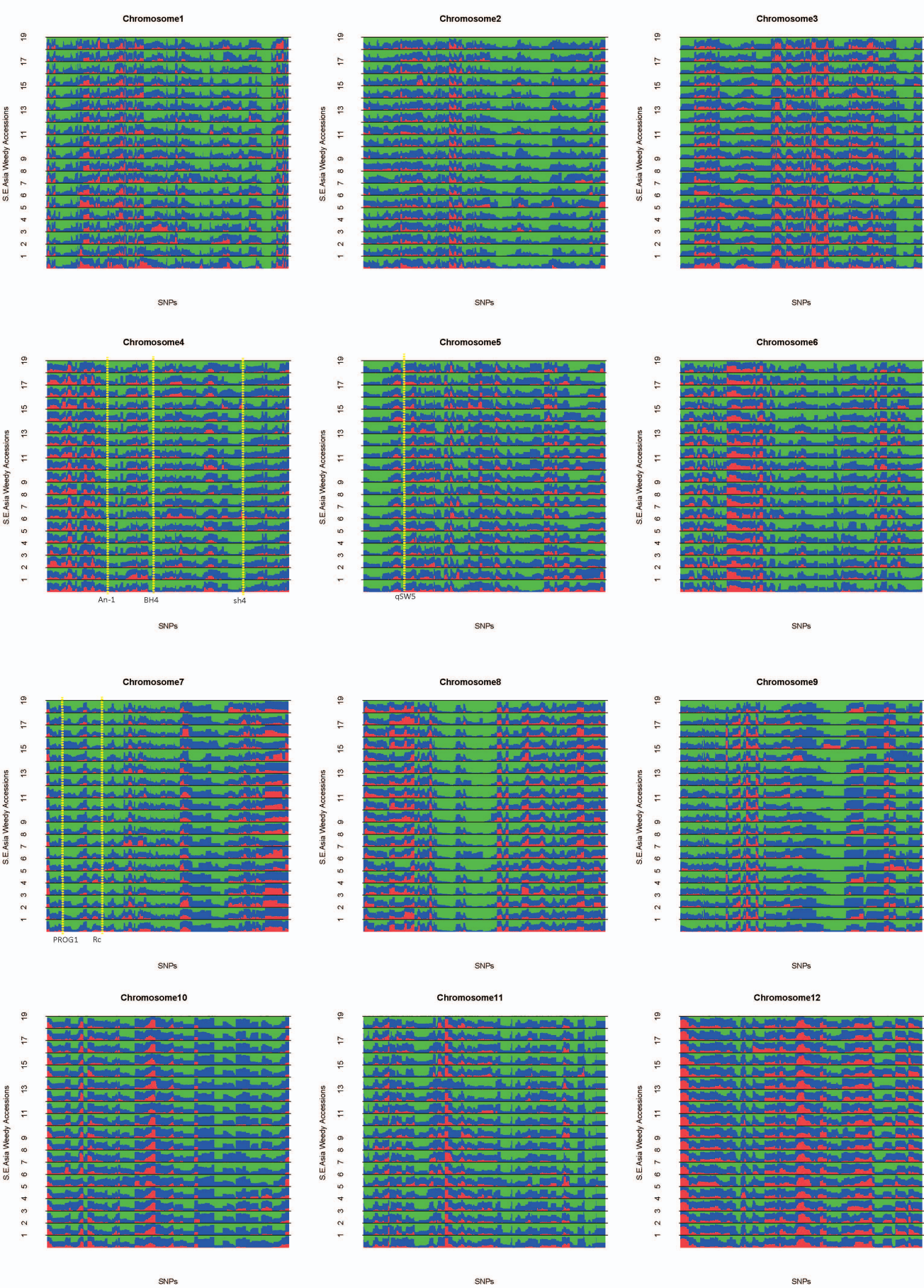

Supplement: Supplementary file 5 [file EVA-12-93-s005.pdf]
